# Long-term preoperative glycemic control restored the perioperative neutrophilic phagocytosis activity in diabetic mice

**DOI:** 10.1186/s12902-020-00629-x

**Published:** 2020-09-29

**Authors:** Daichi Fujimoto, Yuki Nomura, Moritoki Egi, Norihiko Obata, Satoshi Mizobuchi

**Affiliations:** Division of Anesthesiology, Department of Surgery Related, Kobe University Graduate School of Medicine, 7-5-2, Kusunoki-cho, Chuo-ku, Kobe, Hyogo 650-0017 Japan

**Keywords:** Diabetes mellitus, Duration of preoperative glycemic control, Surgery, Neutrophil, Phagocytosis, Reactive oxygen species, Mice

## Abstract

**Background:**

The risk of surgical site infection has been reported to be higher in patients with poorly controlled diabetes. Since chronic hyperglycemia impairs neutrophil functions, preoperative glycemic control may restore neutrophil function. However, long-term insulin therapy may lead to a delay in surgery, which may be a problem, especially in cancer surgery. It is therefore unfortunate that there have been few studies in which the optimal duration of perioperative glycemic control for diabetes with chronic hyperglycemia was investigated. Therefore, we investigated the effects of preoperative long-term insulin therapy and short-term insulin therapy on perioperative neutrophil functions in diabetic mice with chronic hyperglycemia.

**Methods:**

Five-week-old male C57BL/6 J mice were divided into four groups (No insulin (Diabetes Mellitus: DM), Short-term insulin (DM), Long-term insulin (DM), and Non-diabetic groups). Diabetes was established by administrating repeated low-dose streptozotocin. The Short-term insulin (DM) group received insulin therapy for 6 h before the operation and the Long-term insulin (DM) group received insulin therapy for 5 days before the operation. The No insulin (DM) group and the Non-diabetic group did not receive insulin therapy. At 14 weeks of age, abdominal surgery with intestinal manipulation was performed in all four groups. We carried out a phagocytosis assay with fluorescent microspheres and a reactive oxygen species (ROS) production assay with DCFH-DA (2′,7′-dichlorodihydrofluorescein diacetate) before and 24 h after the operation using FACSVerse™ with BD FACSuite™ software.

**Results:**

Blood glucose was lowered by insulin therapy in the Short-term insulin (DM) and Long-term insulin (DM) groups before the operation. Neutrophilic phagocytosis activities before and after the operation were significantly restored in the Long-term insulin (DM) group compared with those in the No insulin (DM) group (before: *p* = 0.0008, after: *p* = 0.0005). However, they were not significantly restored in the Short-term insulin (DM) group. Neutrophilic ROS production activities before and after the operation were not restored in either the Short-term insulin (DM) group or Long-term insulin (DM) group.

**Conclusions:**

Preoperative and postoperative phagocytosis activities are restored by insulin therapy for 5 days before the operation but not by insulin therapy for 6 h before the operation.

## Background

It has been reported that about 10% of patients undergoing elective surgery have diabetes mellitus (DM) [1, 2] and that about 20% of those patients have poor glycemic control preoperatively [2, 3]. The risk of postoperative infectious complications was shown to be significantly higher in patients with diabetes than in patients without diabetes [1]. Chronic hyperglycemia seen in poorly controlled diabetes would inhibit the phosphoinositide 3-kinase (PI3K)-Akt signaling pathway [4], which may be associated with an increase of insulin resistance, and also impairment of neutrophil functions including phagocytosis [5, 6]. Indeed, poorly controlled diabetes was reported to be associated with an additional higher risk of surgical site infection (SSI) [7–9].

Insulin therapy may be beneficial for restoring such an impairment of the PI3K-Akt pathway [10]. Therefore, preoperative glycemic control would improve neutrophil functions in patients with DM, which may result in a reduction in the incidence of SSI. The Centers for Disease Control and Prevention Guideline recommends lowering perioperative blood glucose levels [11], although there have been few studies in which the effect of preoperative insulin therapy in patients with diabetes, especially in those with chronic hyperglycemia, was investigated.

In the ADVANCE study, it was shown that long-term insulin control is beneficial for patients with DM [12]. However, preoperative long-term insulin therapy may lead to a delay in surgery, which may be a problem for some types of surgery, especially cancer surgery. As there have been few studies in which the effects of preoperative short-term insulin therapy and long-term insulin therapy were compared, the optimal duration of preoperative glycemic control in patients with diabetes is still unclear. Considering ethical issues related to a delay of surgery, it might be difficult to perform a clinical study to compare the effects of different durations of preoperative insulin therapy in patients with DM without any clear rationale from basic research.

We therefore conducted a study to assess the effects of preoperative short-term insulin therapy and long-term insulin therapy on perioperative neutrophil functions in diabetic mice with chronic hyperglycemia. Our null hypothesis is that preoperative short-term insulin therapy and long-term insulin therapy have no effect on perioperative neutrophil functions in compared to neutrophil functions with no insulin therapy.

## Methods

**Fig. 1:**
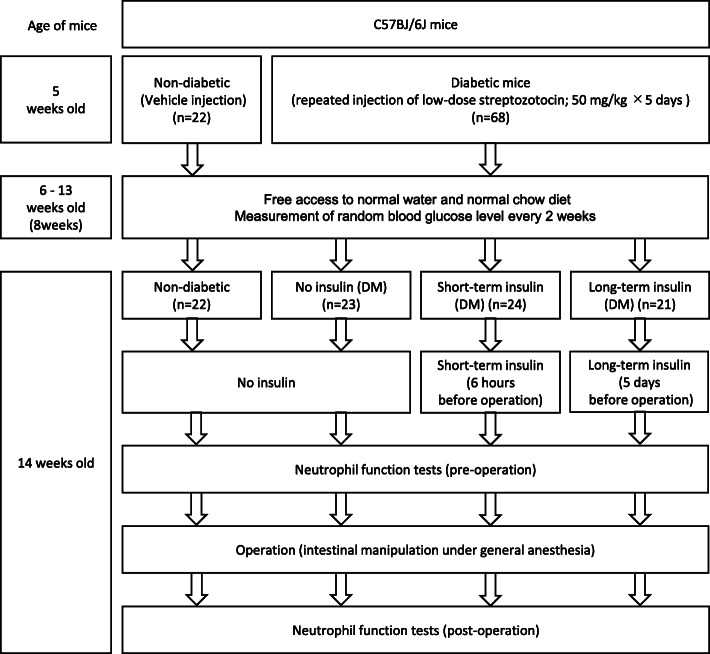
Experimental design flowchart. C57BL/6 J mice were induced DM using repeated injections of low-dose streptozotocin at 5 weeks old. For non-diabetic, mice were administered only a vehicle at the age of 5 weeks. Diabetic mice (*n* = 68) and non-diabetic mice (*n* = 22) were fed for 8 weeks and were measured blood glucose levels in tail vein blood every 2 weeks. Diabetic mice were divided at 14 weeks of age into three groups: No insulin (DM) (*n* = 23), Short-term insulin (DM) (*n* = 24) and Long-term insulin (DM) (*n* = 21) groups. In the No insulin (DM) group, no insulin was administered preoperatively. In the Short-term insulin (DM) and Long-term insulin (DM) groups, neutral protamine hagedorn (NPH) insulin was injected subcutaneously. In the Short-term insulin (DM) group, insulin was injected at 6 h before the operation, and in the Long-term insulin (DM) group, insulin was injected every 12 h for 5 days before the operation. The four groups were performed intestinal manipulation at 14 weeks of age under general anesthesia. Neutrophil functions were examined before and 24 h after the operation by neutrophil phagocytosis assay and neutrophil ROS production assay

This study was approved by the Kobe University Animal Experiment Committee (approved on October 23, 2017, No. P151004). Male C57BL/6 J mice (4 weeks old; body weight, 16–18 g) were purchased from Japan SLC (Shizuoka, Japan). The animals were maintained in a temperature- and humidity-controlled room (22–25 °C, 50–60%) on a 12-h light-dark cycle. They had free access to normal water and normal chow diet (CLEA Rodent Diet CE-2: CLEA Japan, Inc.). The study flow is summarized in Fig. 1.

### Mice with streptozotocin-induced chronic diabetes

To assess the impact of preoperative glucose control in mice with chronic hyperglycemia and to avoid the contribution of an effect related to obesity or leptin abnormality on immune function, we induced DM using repeated injections of low-dose streptozotocin (STZ: Wako Pure Chemical Industries, Osaka, Japan). Mice was intraperitoneally administrated STZ (50 mg/kg body weight) for 5 consecutive days to induce diabetes at the age of 5 weeks according to prior studies [13, 14]. (Fig. 1). For non-diabetic controls, mice were administered only a vehicle at the age of 5 weeks.

Diabetic mice (*n* = 68) and non-diabetic mice (*n* = 22) were fed for 8 weeks. We measured blood glucose levels in tail vein blood using a glucometer (Glutest Neo alfa®, Sanwa Kagaku Kenkyusho, Japan) every 2 weeks (Fig. 1). We defined the development of diabetes as random blood glucose level ≧ 300 mg/dL (17 mM) [15] at the age of 14 weeks.

### Insulin therapy

We divided the diabetic mice at 14 weeks of age into three groups: No insulin (DM) (*n* = 23), Short-term insulin (DM) (*n* = 24) and Long-term insulin (DM) (*n* = 21) groups (Fig. 1). In the No insulin (DM) group, no insulin was administered preoperatively. In the Short-term insulin (DM) and Long-term insulin (DM) groups, neutral protamine hagedorn (NPH) insulin (Humulin N; Eli Lilly, Indiana, USA) was injected subcutaneously to maintain the blood glucose level below 200 mg/dL using an insulin sliding scale according to 12-hourly blood glucose measurements (Supplemental file 1). In the Short-term insulin (DM) group, insulin was injected at 6 h before the operation, and in the Long-term insulin (DM) group, insulin was injected every 12 h for 5 days before the operation.

During the study period, mice had free access to normal water and normal chew diet. We did not use insulin after the operation in this study, because many of the mice in an experimental pilot study suffered from severe hypoglycemia due to the use of insulin after the operation.

Daily insulin sensitivity factor (ISF) as a surrogate of insulin sensitivity [16], which is the drop in blood glucose caused by 1 unit of insulin, was calculated by using the following formula [17]: ISF (mg・dL^− 1^・IU^− 1^) = change in blood glucose (mg/dL) / amount of insulin (IU). Body weight were also measured 5 days before operation and the day of operation.

### Surgical procedure

Considering our clinical question that “how we should control preoperative glycemic control in diabetic patients who are planned common surgery such as colectomy”, we choose an intestinal manipulation as a surgery model [18].

**Fig. 2:**
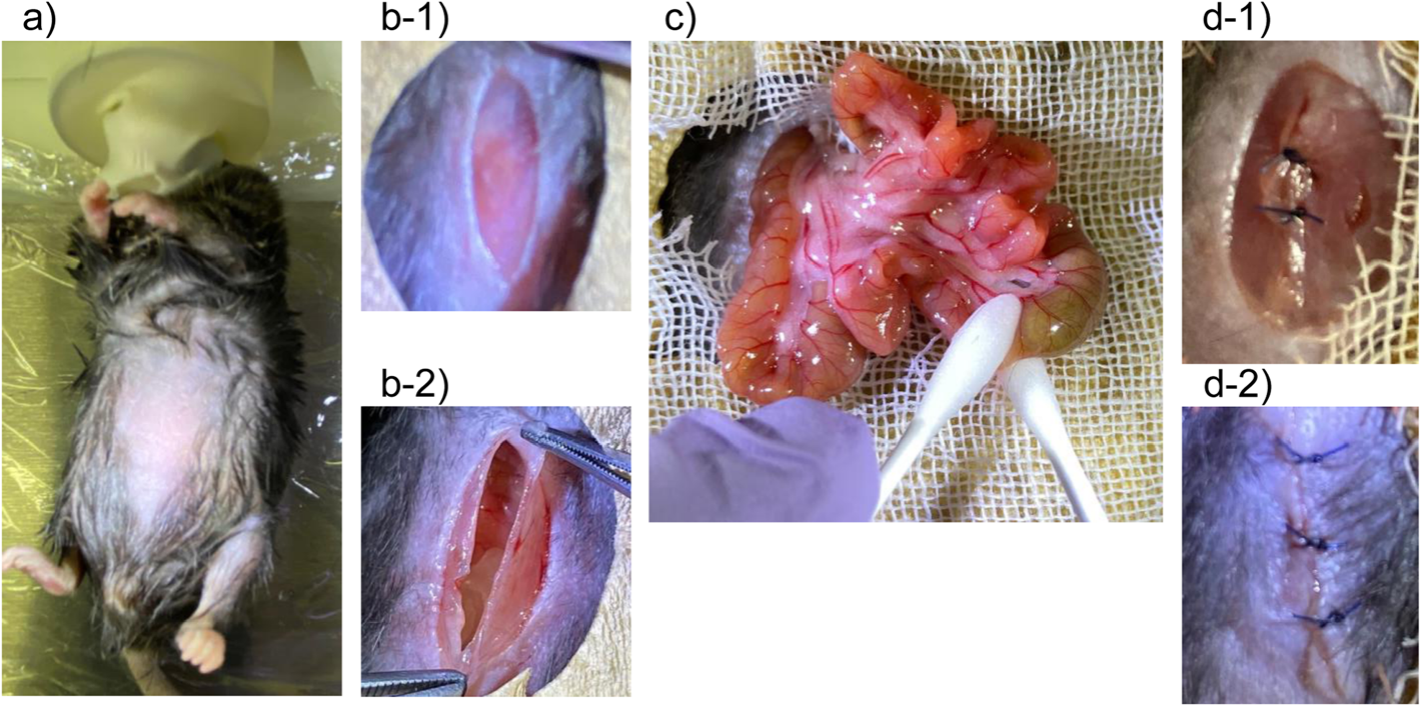
Surgical procedures. **a** A mouse was placed in the supine position on a heating pad (37 °C) and its hair was shaved after induction of general anesthesia using sevoflurane and air. **b-1**, **b-2**) After injection of 1% lidocaine, a vertical incision of 0.5 cm in length was made in the middle of the abdomen. **c** The small bowel luminal contents from the pylorus to the cecum were removed by using two moist and sterile cotton sticks. **d-1**, **d-2**) The surgical wound was closed with 5–0 nylon

We performed intestinal manipulation in all 4 groups, No insulin (DM) group (*n* = 23), Short-term insulin (DM) group (*n* = 24), Long-term insulin (DM) group (*n* = 21) and Non-diabetic group (*n* = 22), at 14 weeks of age under general anesthesia with 3.5% sevoflurane and air, as shown Fig. 2. Each mouse was placed in the supine position on a heating pad (37 °C) during the procedure and its hair was shaved (Fig. 2; a). After injection of 1% lidocaine (Maruishi Pharmaceutical, Osaka, Japan), a vertical incision of 0.5 cm in length was made in the middle of the abdomen (Fig. 2; b-1, b-2). The small bowel luminal contents were moved by using two moist and sterile cotton sticks from the pylorus to the cecum [18] (Fig. 2; c). The surgical wound was closed with 5–0 nylon (Natume Seisakusho Co., Tokyo, Japan) (Fig. 2; d-1, d-2). After the surgical procedure, EMLA® cream including 2.5% lidocaine and 2.5% prilocaine (Sato Pharmaceutical Co, Tokyo, Japan) was applied to the surgical site for analgesia. Each animal was placed under a heating lamp until recovery from anesthesia. After completing the experiment, mice were deeply anesthetized with intraperitoneal administration of pentobarbital (150 mg / kg) and euthanized by cervical dislocation after confirmation of their unconsciousness [19].

### Analysis of neutrophil function

Neutrophil functions were examined before and 24 h after the operation by using two assays. The time point of 24 h after the operation was selected according to results of prior studies showing that the peak of alternation of neutrophil function occurs at 24 h after inducing infection in mice [20] and 24 h after surgery in humans [21]. We assessed neutrophil phagocytosis rate were examined as surrogate of neutrophil functions. A neutrophil phagocytosis assay was carried out by using fluorescently labeled microspheres. Briefly, neutrophils were isolated from peripheral blood after lysis of red blood cells (RBC). The cells were resuspended with RPMI1640 (Gibco® Carisbad, CA, USA) and incubated with Fluoresbrite® Polychromatic Red Microspheres (particles of 2.0 μm in diameter, 2.0 × 10^7^ particles/mL, Polysciences, Inc. Warrington, PA, USA) in RPMI1640 for 2 h in 5% CO_2_ at 37 °C. The cells that phagocytosed microspheres were re-suspended with FACS buffer (PBS (−) including 100 U/ml penicillin, 100 μg/ml streptomycin, 2% FBS, and 2 mM Na_2_EDTA). After incubation with a purified anti-CD16/CD32 antibody (Biolegend®, SanDiego, CA, USA) for blocking Fc receptors, the cells were incubated with Pacific Blue™ anti-mouse Ly-6G (peripheral neutrophil surface marker proteins) antibody (Biolegend®, SanDiego, CA, USA) on ice in the dark. We analyzed the Ly-6G-positive cells as neutrophils with fluorescence of microspheres by using FACSVerse™ with BD FACSuite™ software (BD Bioscience, San Jose, CA, USA). Neutrophil phagocytosis rate was calculated by using following formula: Phagocytosis rate (%) = number of neutrophils with positive fluorescence of microspheres / total number of neutrophils × 100.

An assay of reactive oxygen species (ROS) generated by neutrophils was carried out according to a previous study [22]. Peripheral blood was incubated for 30 min at 37 °C with 5 μM 2′, 7′-dichlorofluorescein-diacetate (DCFH-DA, Merck KGaA, Darmstadt, Germany). Blood samples were incubated with phorbol myristate acetate (PMA: 25 μg/ml, Wako Pure Chemical Industries, Osaka, Japan) for 30 min at 37 °C to stimulate neutrophils. Then samples were placed on ice to stop the reactions. After lysis of RBC, neutrophils were isolated from blood samples and were incubated with purified anti-CD16/CD32 antibody for Fc block and with Pacific Blue™ anti-mouse Ly-6G antibody on ice to stain neutrophils. Green fluorescence intensity of 2′, 7′-dichlorodihydrofluorescein (DCF) in Ly-6G-positive cells was measured using a FACSVerse™. The results are shown as mean fluorescence intensity (MFI).

### Statistical analysis

Data are shown as median values with interquartile range (IQR). Comparisons were performed using the Mann-Whitney U test for unpaired data and Wilcoxon’s signed-rank test for paired data with Prism 8 (GraphPad Software, San Diego, USA).

As our null hypothesis was that preoperative short-term insulin therapy and long-term insulin therapy have no effect on perioperative neutrophil functions in compared to neutrophil functions with no insulin therapy, we considered the No insulin (DM) group as a reference for comparisons among the four groups. Considering the bias of multiple comparisons (3 times), a *p*-value < 0.0167 was considered to indicate statistical significance for the analysis of blood glucose levels and results of neutrophil function tests. For comparisons of insulin demand, ISF value, we considered the value on the first day in the Long-term insulin (DM) group as a reference. For this analysis, a p-value < 0.01 was considered to indicate statistical significance for adjusting the bias of multiple comparisons (5 times).

To calculate the sample size for the current study, we considered an absolute difference of 10% in the phagocytosis rate and 500 in ROS to be meaningful. Assuming standard deviations of 8% for phagocytosis rate and 250 for ROS, an α level of 0.0167 and a power of 0.80, approximately 15 mice and 6 mice were required in each cohort.

## Results

### Perioperative blood glucose levels

**Fig. 3:**
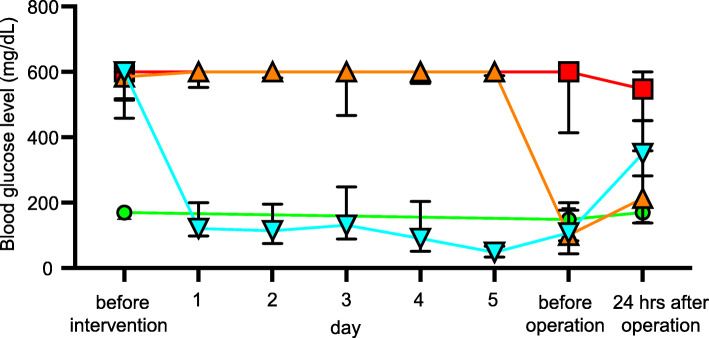
Perioperative blood glucose levels in the four groups. Data are presented as median values and interquartile range (mg/dL). The red boxes and red line indicate mean blood glucose level in the No insulin (DM) group. The orange triangles and orange line indicate mean blood glucose level in the Short-term insulin (DM) group. The blue inversed triangles and blue line indicate mean blood glucose level in the Long-term insulin (DM) group. The green circles and green line indicate mean blood glucose level in the Non-diabetic group

Figure 3 shows perioperative blood glucose levels. In the Non-diabetic group (green), blood glucose levels were within the normal range throughout the perioperative period. In the No insulin (DM) group (red), blood glucose level was around 600 mg/dL throughout the perioperative period. In the Short-term insulin (DM) group (orange), blood glucose level was around 600 mg/dL for 5 preoperative days and then decreased before and after the operation. In the Long-term insulin (DM) group (blue), blood glucose level was around 600 mg/dL before insulin therapy and then decreased after commencement of insulin therapy. The blood glucose levels before and after the operation in the No insulin (DM) group were significantly higher than the levels in the other three groups. The blood glucose levels before and after the operation in the Short-term insulin (DM) group were not significantly different from those in the Long-term insulin (DM) group.

### Insulin sensitivity factors and body weight during the insulin therapy period

On the first day, the median insulin dose in the Long-term insulin (DM) group was 7 IU (IQR: 4.5–10), which was not significantly different from the median dose of 6 IU (IQR: 5.25–10) in the Short-term insulin (DM) group (*p* = 1.00). In the Long-term insulin (DM) group, the insulin dose required to control glucose levels gradually decreased, and the doses were significantly lower on the 3rd, 4th and 5th days of insulin therapy than on the first day (3rd, 4th, and 5th days vs. first day: *p* = 0.0001, < 0.0001, and < 0.0001, respectively).

**Fig. 4:**
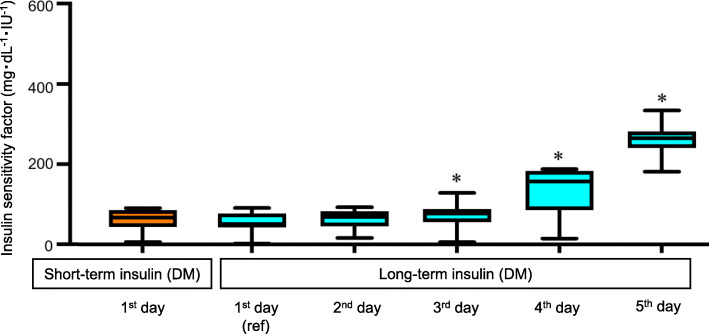
Changes in insulin sensitivity factor (ISF) during insulin therapy. Orange box plot shows ISF in the Short-term insulin (DM) group. Blue box plots show ISF in the Long-term insulin (DM) group for 5 days. The ISF on each day is compared with the value on the first day in the Long-term insulin (DM) group. *p < 0.01 (as five comparisons). Data are presented as median values and interquartile range (mg・dL^− 1^・IU^− 1^)

Figure 4 shows the ISFs in the Long-term insulin (DM) and Short-term insulin (DM) groups during the insulin therapy period. The median ISF on the first day in the Long-term insulin (DM) group was 51.3 mg・dL^− 1^・IU^− 1^ (IQR: 43.1–76.1), which was not significantly different from the median ISF of 67 mg・dL^− 1^・IU^− 1^ (IQR: 46.7–83.3) in the Short-term insulin (DM) group (*p* = 0.47). In the Long-term insulin (DM) group, ISF gradually increased and was significantly higher on the 3rd, 4th and 5th days of insulin therapy than on the first day (3rd, 4th, and 5th days vs. first day: *p* = 0.0061, 0.0004, and < 0.0001, respectively).

Supplemental file 2 shows body weight 5 days before operation and the day of operation. In the Long-term insulin (DM) group and No insulin (DM) group, there was significant difference in body weight between 5 days before operation and the day of operation.

### Phagocytosis activities before and after the operation

**Fig. 5:**
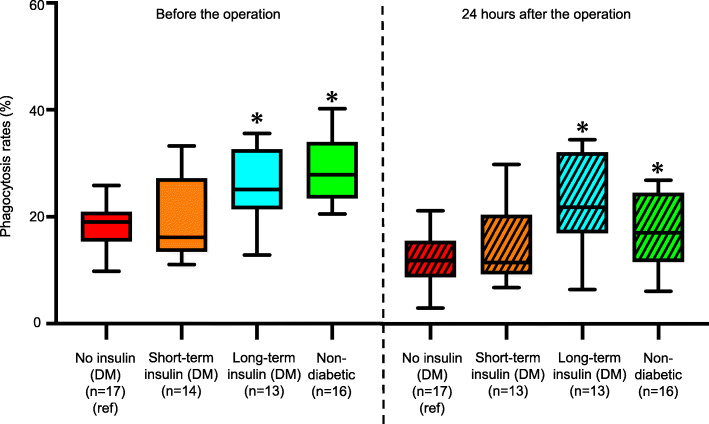
Neutrophil phagocytosis rates before and 24 h after the operation in the four groups. Left box plots show neutrophil phagocytosis rates before the operation. Right box plots show the rates 24 h after the operation. We used the neutrophil phagocytosis rate in the No insulin (DM) group (red) as a reference and compared it with the rates in the Short-term insulin (DM) group (orange), Long-term insulin (DM) group (blue) and Non-diabetic group (green). *p < 0.0167 (as three comparisons). Data are presented as median values and interquartile range (%)

Figure 5 (left side) shows the phagocytosis rates before the operation in the four groups. In the Short-term insulin (DM) group, the median preoperative phagocytosis rate was 16.2% (IQR: 13.6–25.6), which was not significantly different from the median rate of 19.0% (IQR: 16.8–21.0) in the No insulin (DM) group (*p* = 0.87). In the Long-term insulin (DM) group, the median phagocytosis rate was 25.1% (IQR: 21.5–30.1), which was significantly higher than that in the No insulin (DM) group (*p* = 0.0008). The median phagocytosis rate in the Long-term insulin (DM) group was comparable to the median rate of 27.9% (IQR: 23.3–34.2) in the Non-diabetic group (*p* = 0.63). Figure 5 (right side) shows the phagocytosis rates 24 h after the operation in the four groups. The trends were similar to those before the operation. In the Short-term insulin (DM) group, the median postoperative phagocytosis rate was 11.7% (IQR: 9.8–20.6), which was not significantly different from the median rate of 12.1% (IQR: 8.8–15.1) in the No insulin (DM) group (*p* = 0.41). In the Long-term insulin (DM) group, the median postoperative phagocytosis rate was 22.2% (IQR: 18.3–32.0), which was significantly higher than that in the No insulin (DM) group (*p* = 0.0005).

The median phagocytosis rate in the Long-term insulin (DM) group was comparable to the median rate of 17.3% (IQR: 11.7–25.1) in the Non-diabetic group (*p* = 0.14).

Supplemental file 3 show the proportion of neutrophils in the blood samples and the neutrophil count in the peripheral blood before and after operation in each group. Percentage of neutrophils before operation in the Non-diabetic group was significantly lower than those in the No insulin (DM) group. After operation, neutrophil count was almost doubled in the No insulin (DM), Short-term insulin (DM) and Non-diabetic groups in compared with before operation. However, in the Long-term insulin (DM), there is no significant difference of neutrophil count between before and after operation. Supplemental file 4 shows neutrophil count according to the number of phagocytosis beads and the total count of phagocytosed beads before (upper) and after (lower) operation in each group. The median total count of phagocytosed beads before operation was 4222 (IQR:3013–5984) in the Short-term insulin (DM), 12,142 (IQR:6025–15,332) in the Long-term insulin (DM) and 8238 (IQR:4976–12,550) in the Non-diabetic group, which were not significantly differed with those of 6275 (IQR:3628–8188) in the No insulin (DM) group (*p* = 0.084,0.043 and 0.074, respectively). There is significant difference between the Short-term insulin (DM) and Long-term insulin (DM). The median total count of phagocytosed beads after operation was 7351 (IQR:2012–20,808) in the Short-term insulin (DM), 9764 (IQR:5446–19,605) in the Long-term insulin (DM) and 9869 (IQR:7767–19,560) in the Non-diabetic group, which were not significantly differed with those of 7177 (IQR:3528–10,219) in the No insulin (DM) group (*p* = 0.84,0.43 and 0.021, respectively).

### ROS production activities before and after the operation

**Fig. 6:**
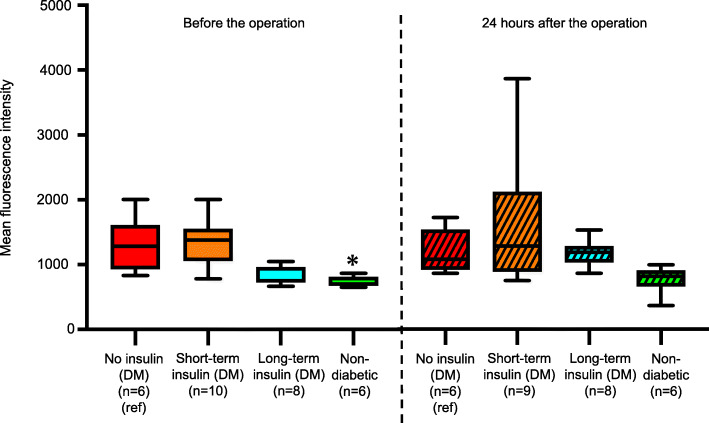
Neutrophil ROS production before and 24 h after the operation in the four groups. Left box plots show neutrophil ROS production levels before the operation. Right box plots show neutrophil ROS production levels 24 h after the operation. We used neutrophil ROS production in the No insulin (DM) group (red) as a reference and compared it with ROS production levels in the Short-term insulin (DM) group (orange), Long-term insulin (DM) group (blue) and Non-diabetic group (green). *p < 0.0167 (as three comparisons). Data are presented as median values and interquartile range

Figure 6 (left) shows the MFIs as measures of ROS production activity before the operation in the four groups. In the No insulin (DM) group, the median MFI was 1282 (IQR: 920–1623), which was not significantly different from the median value of 1381 in the Short-term insulin (DM) group (IQR: 1043–1565, *p* = 0.90) or the median value of 935 in the Long-term insulin (DM) group (IQR: 716–974, *p* = 0.059). The median MFI in the Non-diabetic group was 704 (IQR: 665–823), which was significantly different from that in the No insulin (DM) group (*p* = 0.0043). Figure 6 (right) shows the MFIs 24 h after the operation in the four groups. The trends were similar to those before the operation. In the No insulin (DM) group, the median MFI was 1093 (IQR: 918–1574), which was not significantly different from the median value of 1303 in the Short-term insulin (DM) group (IQR: 888–2165, *p* = 0.78) or the median value of 1201 in the Long-term insulin (DM) group (IQR: 1032–1311, *p* = 0.64). The median MFI in the Non-diabetic group was 829 (IQR: 655–930), which was not significantly different from that in the No insulin (DM) group (*p* = 0.026). Supplemental Fig. 5 shows the representative ROS image of flowcytometry in each group.

## Discussion

### Key findings

The aim of the present study using mice with induced by repeated injections of low-dose STZ chronic diabetes was to determine the impact of the duration of preoperative insulin therapy on phagocytosis activity and ROS production activity of neutrophils before and after an operation. In our study, chronic hyperglycemia lasting for about 8 weeks was associated with a 40% reduction in neutrophil phagocytosis activity. Surgical stress induced a further 20–40% suppression of neutrophil phagocytosis activity in all four groups. We found that impaired phagocytosis activity induced by chronic diabetes was restored to a level similar to that in the Non-diabetic group by preoperative insulin therapy for 5 days but not by preoperative insulin therapy for 6 h. Such a difference between phagocytosis activities with short-term and long-term preoperative insulin therapy was observed without a significant difference in blood glucose levels immediately before and 24 h after the operation.

### Related animal studies

The literature includes two prior relevant studies. Yano et al. conducted a study using 16-week-old diabetic *db/db* mice and high-fat diet-fed mice [23]. Blood glucose levels in their diabetic mice were approximately between 216 and 252 mg/dL. Blood glucose level gradually decreased with insulin treatment and was > 126 mg/dL after 7 days of insulin therapy. Their insulin treatment significantly improved preoperative phagocytosis activity of neutrophils and decreased the maximal diameter of surgical site infection in both types of mice. Insulin treatment significantly increased superoxide production in *db/db* mice but decreased it in high-fat diet-fed mice. They did not assess neutrophilic function after the operation. We found that long-term preoperative insulin therapy improved preoperative phagocytosis activity of neutrophils, being in agreement with the results of Yano’s study. The novelty of our study is that it showed the effect of preoperative insulin therapy on phagocytosis activity of neutrophils that lasted for up to 24 h after the operation, and such a recovery seen in the Long-term insulin (DM) group was significant compared with that the Short-term insulin (DM) group.

Kroin et al. conducted a study using Sprague-Dawley rats with diabetes induced by STZ [24]. Blood glucose levels in their rats were over 250 mg/dL. The long-term insulin therapy group received insulin treatment for 2 weeks and the short-term insulin therapy group received insulin treatment just before the operation. Both groups achieved a normal blood glucose level on day 3 and day 6 after operation, and the bacterial burden in the biceps femoris muscle was reduced compared to that with no glycemic control. Regarding the effect of preoperative short-term insulin therapy, the result of our study is conflict with the results of Kroin’s study. The differences in results might come from the following notable points in Kroin’s study: blood glucose levels for 6 days after surgery were equally controlled in both the short-term and long-term groups, and the period of chronic hyperglycemia was relatively short (3 weeks) for the long-term group. It is possible that both the preoperative insulin therapy and postoperative insulin therapy contributed to their results.

### Interpretation of our findings

Our findings and the results of the above-described relevant studies might translate to human diabetic patients and generate the hypothesis that preoperative glycemic control influences postoperative outcomes in diabetic patients with chronic hyperglycemia. Our results obtained for the Long-term insulin (DM) group are in agreement with the results of the above-described studies [23, 24]. However, short-term insulin therapy did not significantly improve neutrophil phagocytosis activity. There are several possible explanations for this finding.

First, inhibition of the PI3K-Akt signaling pathway by chronic hyperglycemia has been reported to contribute to the suppression of neutrophil phagocytosis activity [4, 10]. Such an impairment of the PI3K-Akt pathway is also associated with deterioration of insulin resistance, which may be restored by insulin therapy [10]. In the current study, ISF gradually improved and the improvement reached statistical significance after insulin therapy for 3 or more days. These results suggest that restoration of insulin sensitivity though various mechanisms including the PI3K-Akt pathway may require a certain duration of insulin therapy rather than single insulin administration. Since the PI3K-Akt pathway contributes to both insulin resistance and neutrophil phagocytosis, our diabetic model may require long-term insulin therapy for a significant improvement of neutrophil phagocytosis activity. Second, in mouse bone marrow, promyelocytes grow into mature neutrophils during a period of 5 days. The mature neutrophils are pooled in bone marrow for 2 days and then released into blood. Finally, neutrophils end their life within 6 h [25]. Since hyperglycemia would influence the glucose level in bone marrow, insulin therapy for 5 days may improve growth circumstances of mature neutrophils in bone marrow and then may contribute to the improvement of neutrophil phagocytosis.

In prior clinical studies, intensive insulin therapy for 2 or 3 weeks was shown to improve insulin sensitivity [26, 27]. Results of radionuclide studies suggested that about 11–12 days are necessary for the transition from myeloblasts to mature neutrophils in bone marrow [28]. If the above mechanisms contribute to the difference in restoration of neutrophil phagocytosis between long-term and short-term preoperative insulin therapy, one may speculate that approximately 2 or 3 weeks is the duration of good glycemic control needed in humans. Since no clinical study has been carried out to assess this concept, it is definitely necessary to conduct future studies to refute or confirm this hypothesis.

Our study had conducted to assess the neutrophil phagocytosis rate as primary outcome of phagocytosis activities. There was significant improvement in the phagocytosis rate 24 h after operation in the Long-term insulin (DM) group compared with the No insulin (DM) group, but not in the Short-term insulin (DM) group. This fact might suggest that the phagocytosis “efficiency” per neutrophil after operation would be better in the Long-term insulin (DM) group. We should note that the total number of phagocytosed beads after operation in the Long-term insulin (DM) group was not significantly differed with those of the No insulin (DM) group or Short-term insulin (DM) group, as neutrophil counts was doubled in the Short-term insulin (DM) and No insulin (DM) groups, whereas they did not increase in the Long-term insulin (DM) group. This finding might suggest that the increase of number of neutrophils in the Short-term insulin (DM) and No insulin (DM) groups would counterbalance the reduction of phagocytosis efficiency, which result in similar number of phagocytosed beads after operation among 4 groups. If this hypothesis is true, this fact would be in same line of the finding of study conducted by Kroin et al. [24].

### Perioperative ROS production activity of neutrophils in diabetic mice and impact of preoperative insulin therapy

Hyperglycemia has been reported to induce activation of the protein kinase C pathway [10] and advanced glycation end-products pathway [29], which may result in an increase in the production of ROS in neutrophils [30, 31]. As was observed in *db/db* mice in Yano’s study [23], the No insulin (DM) group in our study had significantly greater production of ROS than that in the Non-diabetic group before operation.

Although there was a trend for improvement in the production of ROS before operation in the Long-term insulin (DM) group, short-term and long-term insulin treatment had no significant effect on perioperative neutrophilic ROS production in our study. The effect of insulin therapy on perioperative production of ROS may be influenced by various factors including the cause of diabetes [23]. Further examination is needed to clarify the relationship between duration of insulin therapy and changes in production of ROS by neutrophils.

### Limitations

Several limitations of our study need to be considered. First, we did not perform additional insulin therapy after the operation, which may have contributed to our results. Second, we used mice with DM that was induced by using repeated low-dose STZ injections to avoid the contribution of an effect related to obesity or leptin abnormality on immune function. However, considering the high prevalence of type II DM, a future study should be conducted to assess the generalizability of our findings into a type II DM model. Third, we selected 24 h after the operation as the time point for evaluating postoperative neutrophil function according to prior studies [20, 21] and choose an intestinal manipulation as a surgery model. Although we observed that there is no significant difference of neutrophil phagocytosis rates before and 24 h after the operation in each group, this finding might be differed in different time point and different surgery model. Since the time trend of neutrophil function should be relevant, a future study with observations at multiple time points during the postoperative period should be conducted. Furthermore, there is weak generalizability of our finding to moderate or severe surgical stress. Fourth, we did not conduct a study in a group with sham surgery (surgery but not bowel manipulation) or a group with just general anesthesia. Further study including these groups is needed to investigate the effects of bowel manipulation, surgical incision and general anesthesia. Finally, we did not assess the effects of hyperglycemia and insulin therapy on intracellular signaling. The detailed signaling pathways involved in the restoration of neutrophil phagocytosis with different durations of insulin therapy remain to be identified.

## Conclusion

In our model in which chronic hyperglycemia was sustained for 8 weeks, preoperative and postoperative phagocytosis activities of neutrophils were restored by insulin therapy for 5 days before the operation but not by insulin therapy for 6 h before the operation.

## Supplementary information

**Additional file 1: Supplemental file 1.** Insulin sliding scales for the Short-term insulin (DM) and Long-term insulin (DM) groups. **Supplemental file 2.** Preoperative body weight change in each group. This table show preoperative body weight 5 days before operation and the day of operation in each group. Data are presented as median values and interquartile range (g). **Supplemental file 3.** The proportion of neutrophils in the blood samples and the neutrophil counts in the peripheral blood before and after operation in each group. *p < 0.0167 (as three comparisons). Data are presented as median values and interquartile range. **Supplemental file 4.** Neutrophil count according to the number of phagocytosis beads and the total count of phagocytosed beads before and after operation in each group. *p < 0.0167 (as three comparisons). Data are presented as median values and interquartile range. **Supplemental figure 5.** The representative ROS image of flowcytometry in each group.

## Data Availability

The datasets used and/or analyzed during the current study are available from the corresponding author on reasonable request.

## Abbreviations

DM: Diabetes mellitus
ADVANCE: Action in Diabetes and Vascular disease: PreterAx and DiamicroN Controlled Evaluation
STZ: Streptozotocin
NPH: Neutral protamine hagedorn
ISF: Insulin sensitivity factor
RBC: Red blood cells
FACS: Fluorescence-activated cell sorting
PBS: Phosphate buffered saline
ROS: Reactive oxygen species
DCFH-DA: 2′, 7′-dichlorofluorescein-diacetate
PMA: Phorbol myristate acetate
DCF: 2′, 7′-dichlorodihydrofluorescein
MFI: Mean fluorescence intensity
IQR: Interquartile range
PI3K: Phosphoinositide 3-kinase
PKC: Protein kinase C
AGEs: Advanced glycation end-products
