# Supplementary material for: Long-term preoperative glycemic control restored the perioperative neutrophilic phagocytosis activity in diabetic mice
Source: BMC Endocr Disord. 2020 Sep 29;20:146. doi: 10.1186/s12902-020-00629-x (PMC7525964; doi:10.1186/s12902-020-00629-x)
Supplement: Supplementary file 1 — Additional file 1: Supplemental file 1. Insulin sliding scales for the Short-term insulin (DM) and Long-term insulin (DM) groups. Supplemental file 2. Preoperative body weight change in each group. This table show preoperative body weight 5 days before operation and the day of operation in each group. Data are presented as median values and interquartile range (g). Supplemental file 3. The proportion of neutrophils in the blood samples and the neutrophil counts in the peripheral blood before and after operation in each group. *p < 0.0167 (as three comparisons). Data are presented as median values and interquartile range. Supplemental file 4. Neutrophil count according to the number of phagocytosis beads and the total count of phagocytosed beads before and after operation in each group. *p < 0.0167 (as three comparisons). Data are presented as median values and interquartile range. Supplemental figure 5. The representative ROS image of flowcytometry in each group. [file 12902_2020_629_MOESM1_ESM.pptx]

## Slide 1
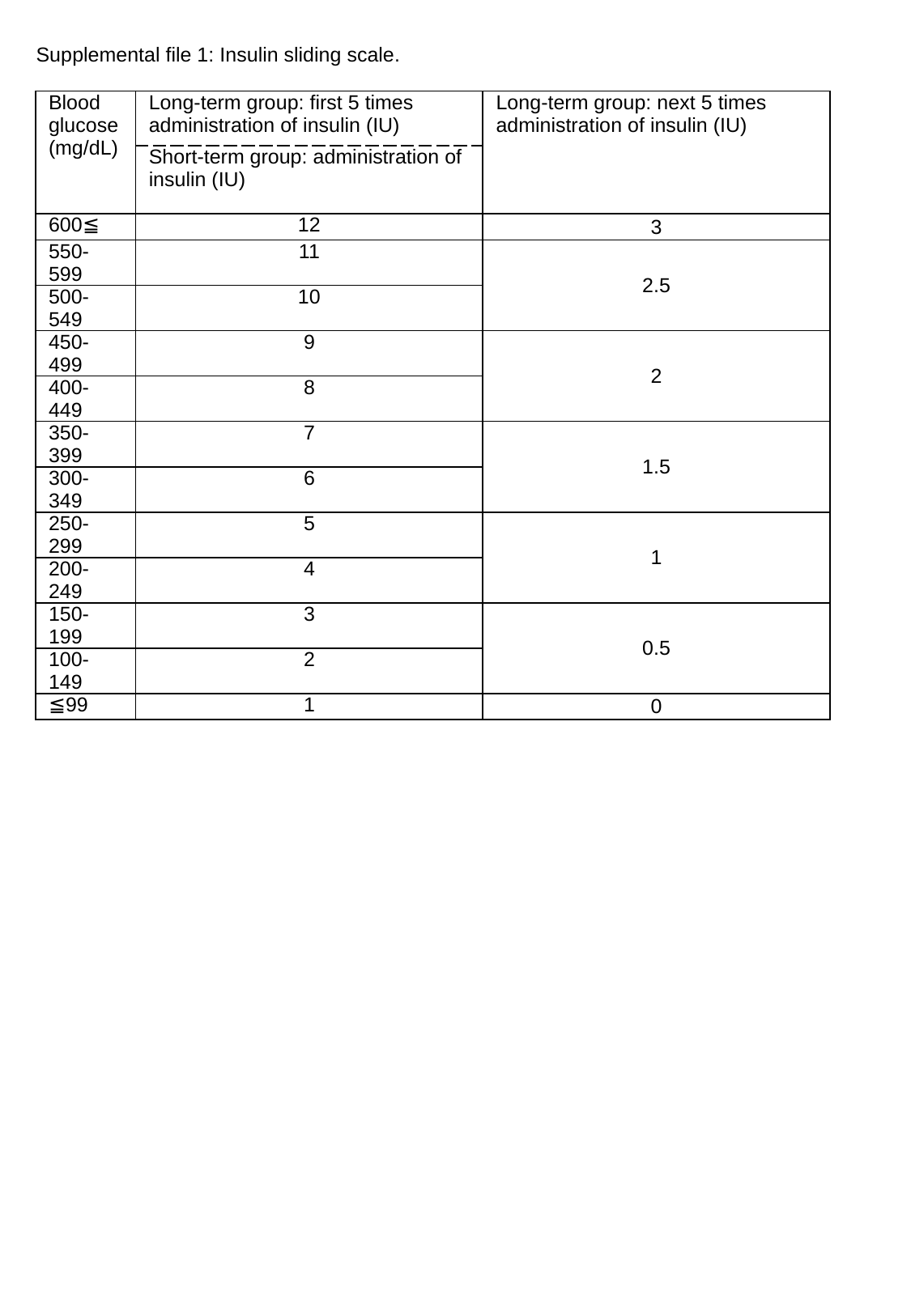

Supplemental file 1: Insulin sliding scale.
| Blood glucose (mg/dL) | Long-term group: first 5 times administration of insulin (IU) | Long-term group: next 5 times administration of insulin (IU) |
| --- | --- | --- |
| | Short-term group: administration of insulin (IU) | |
| 600≦ | 12 | 3 |
| 550-599 | 11 | 2.5 |
| 500-549 | 10 | |
| 450-499 | 9 | 2 |
| 400-449 | 8 | |
| 350-399 | 7 | 1.5 |
| 300-349 | 6 | |
| 250-299 | 5 | 1 |
| 200-249 | 4 | |
| 150-199 | 3 | 0.5 |
| 100-149 | 2 | |
| ≦99 | 1 | 0 |

## Slide 2
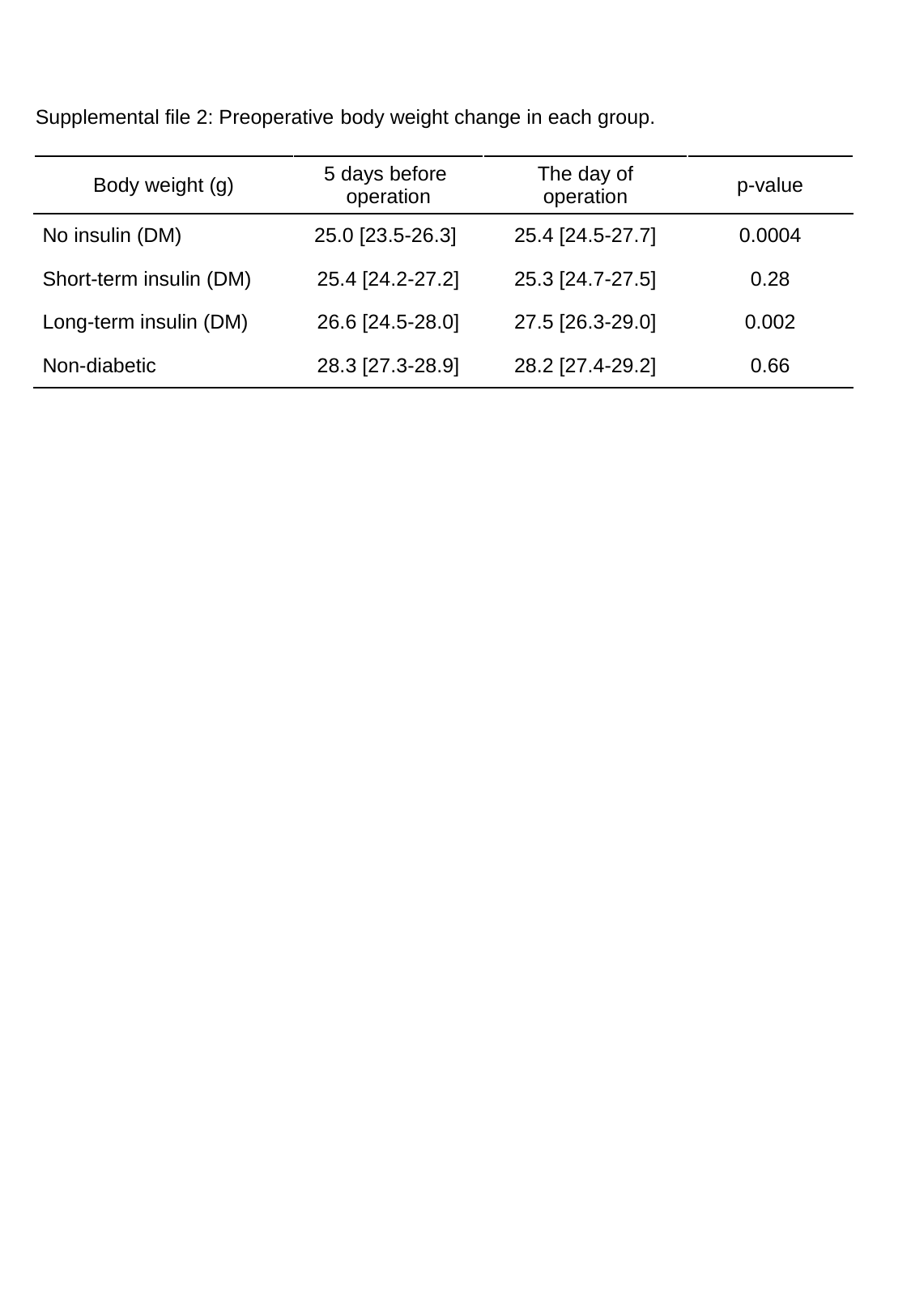

Supplemental file 2: Preoperative body weight change in each group.
| Body weight (g) | 5 days before operation | The day of operation | p-value |
| --- | --- | --- | --- |
| No insulin (DM) | 25.0 [23.5-26.3] | 25.4 [24.5-27.7] | 0.0004 |
| Short-term insulin (DM) | 25.4 [24.2-27.2] | 25.3 [24.7-27.5] | 0.28 |
| Long-term insulin (DM) | 26.6 [24.5-28.0] | 27.5 [26.3-29.0] | 0.002 |
| Non-diabetic | 28.3 [27.3-28.9] | 28.2 [27.4-29.2] | 0.66 |

## Slide 3
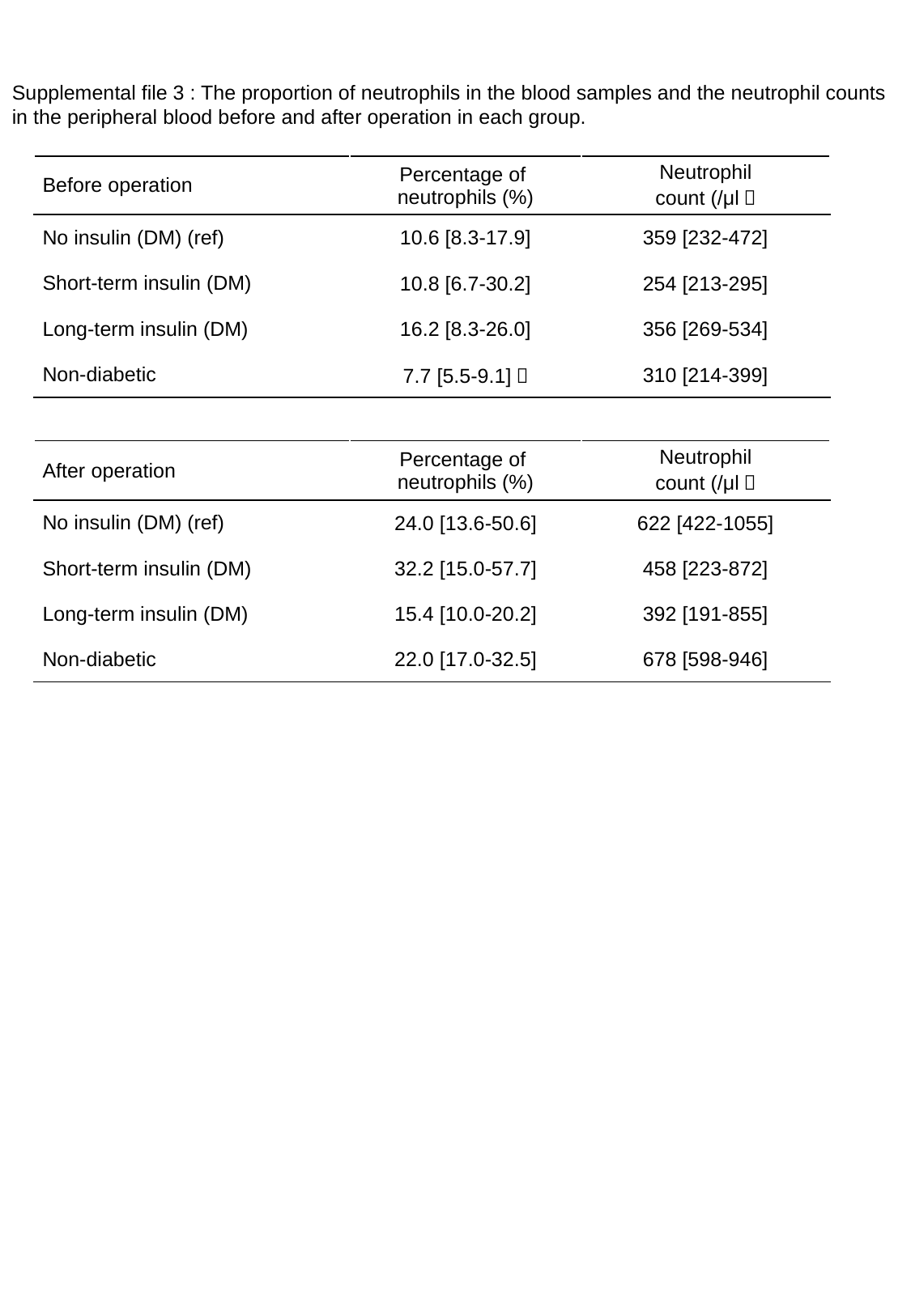

Supplemental file 3 : The proportion of neutrophils in the blood samples and the neutrophil counts in the peripheral blood before and after operation in each group.
| Before operation | Percentage of neutrophils (%) | Neutrophil count (/μl） |
| --- | --- | --- |
| No insulin (DM) (ref) | 10.6 [8.3-17.9] | 359 [232-472] |
| Short-term insulin (DM) | 10.8 [6.7-30.2] | 254 [213-295] |
| Long-term insulin (DM) | 16.2 [8.3-26.0] | 356 [269-534] |
| Non-diabetic | 7.7 [5.5-9.1]＊ | 310 [214-399] |
| After operation | Percentage of neutrophils (%) | Neutrophil count (/μl） |
| --- | --- | --- |
| No insulin (DM) (ref) | 24.0 [13.6-50.6] | 622 [422-1055] |
| Short-term insulin (DM) | 32.2 [15.0-57.7] | 458 [223-872] |
| Long-term insulin (DM) | 15.4 [10.0-20.2] | 392 [191-855] |
| Non-diabetic | 22.0 [17.0-32.5] | 678 [598-946] |

## Slide 4
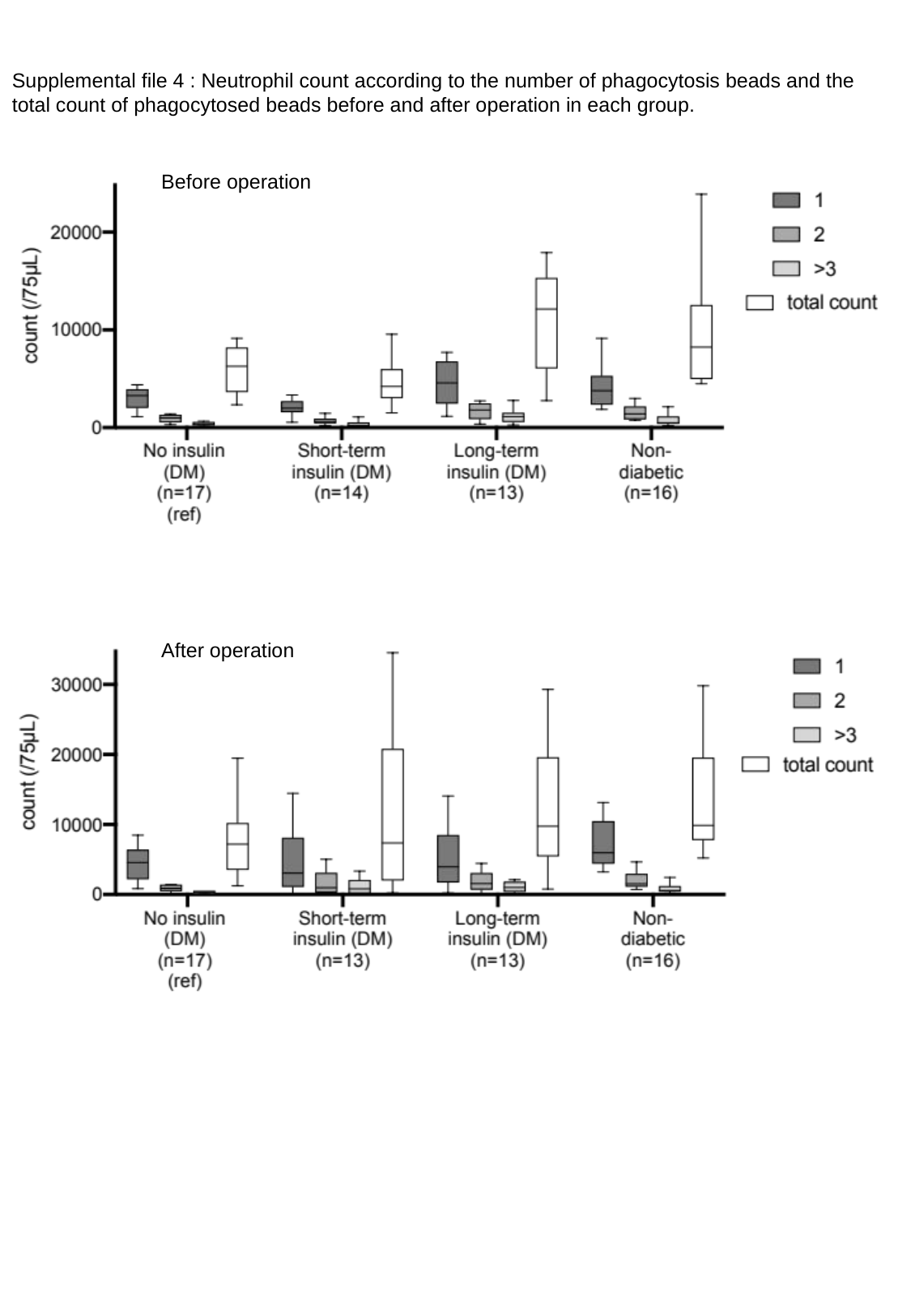

Supplemental file 4 : Neutrophil count according to the number of phagocytosis beads and the total count of phagocytosed beads before and after operation in each group.
Before operation
After operation

## Slide 5
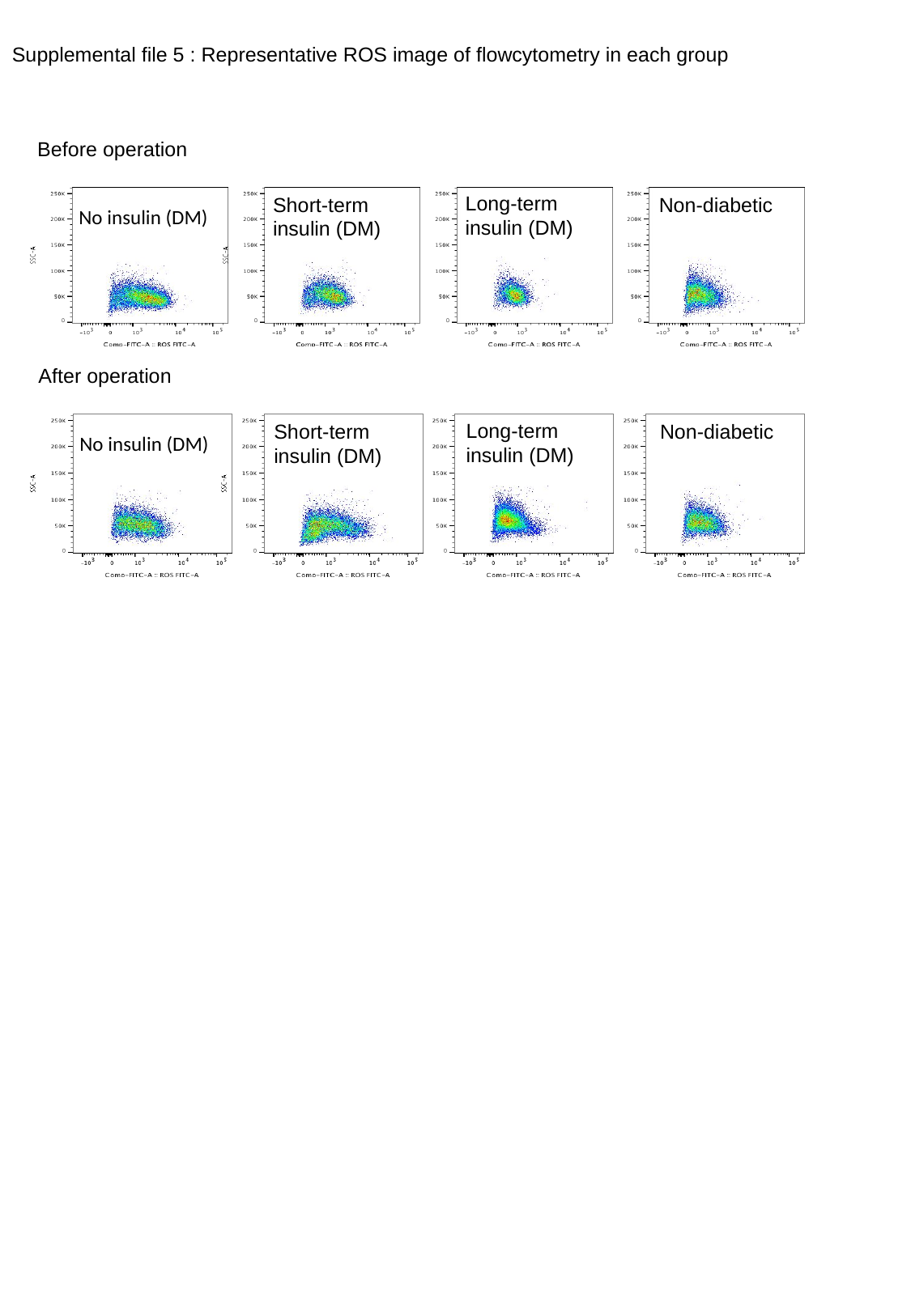

Supplemental file 5 : Representative ROS image of flowcytometry in each group
Before operation
Long-term
insulin (DM)
Short-term
insulin (DM)
Non-diabetic
No insulin (DM)
After operation
Long-term
insulin (DM)
Short-term
insulin (DM)
Non-diabetic
No insulin (DM)
